# Design of novel highly sensitive sensors for crack detection in metal surfaces: theoretical foundation and experimental validation

**DOI:** 10.1038/s41598-023-45556-8

**Published:** 2023-10-29

**Authors:** Zahra Shaterian, Ali K. Horestani, Ferran Martín, Michal Mrozowski

**Affiliations:** 1 Department of Electrical Engineering, Technical and Vocational University (TVU), 14357-61137 Tehran, Iran; 2 Department of Microwave and Antenna Engineering, Faculty of Electronics, Telecommunications, and Informatics, Gdansk University of Technology, Narutowicza 11/12, 80-233 Gdańsk, Poland; 3 Wireless Telecommunication Group, Khayyam Research Institute, Ministry of Science, Research and Technology, Tehran, 64891 Iran; 4 CIMITEC, Departament d’Enginyeria Electrònica, Universitat Autònoma de Barcelona, Bellaterra, Spain

**Keywords:** Electrical and electronic engineering, Design, synthesis and processing

## Abstract

The application of different types of microwave resonators for sensing cracks in metallic structures has been subject of many studies. While most studies have been focused on improving the sensitivity of planar crack sensors, the theoretical foundation of the topic has not been treated in much detail. The major objective of this study is to perform an exhaustive study of the principles and theoretical foundations for crack sensing based on planar microwave resonators, especially defective ground structures (DGS) including complementary split ring resonators (CSRRs). The analysis is carried out from the equivalent circuit model as well as the electromagnetic (EM) field perspectives, and guidelines for the design of crack sensors with high sensitivity are developed. Numerical and experimental validation of the provided theoretical analysis is another aim of this article. With this aim, the developed guidelines are used to design a crack sensor based on a single-ring CSRR. It is shown that the sensitivity of the proposed sensor is almost three times higher than the sensitivity of a conventional double-ring CSRR. Moreover, it is demonstrated that folded dumbbell-shape DGS resonators can be used to achieve even higher sensitivities. The CSRR-based crack sensors presented in this study and other studies available in the literature are only sensitive to cracks with a specific orientation. To address this limitation, a modified version of the DGS is proposed to sense cracks with arbitrary orientations at the cost of lower sensitivity. The performance of all the presented sensors is validated through EM simulation, equivalent circuit model extraction, and measurement of the fabricated prototypes.

## Introduction

Aging and overloading of metallic structures such as steel bridges, oil and gas vessels, power plant equipment, and aircraft fuselages, especially in extreme environments, may lead to fatigue or failure, and eventually catastrophic disasters. The issue usually starts with small cracks on the surface of the metallic structures. Therefore, regular monitoring of metallic structures in terms of small surface cracks is essential^1–5^. For this purpose, various non-destructive techniques based on acoustic, ultrasonic, and eddy current principles have been proposed^6–8^. Despite advantages such as simplicity, low cost, and effectiveness in ideal conditions, these methods may not be efficient in practical situations where sub-millimeter cracks are concealed with dirt and dust or may be covered with a dielectric coating or paint.

To address this issue, microwave technology has been explored for application in metal crack detection^1,9–12^. Among various methods of microwave sensing, sensing based on microwave resonators is perhaps the most widely used method that benefits from high sensitivity, relatively simple application, and scalability to other frequencies in the electromagnetic spectrum. In this method, usually, variations of the resonance frequency of a microwave resonator due to changes in the variable to be sensed are considered as the output variable of the sensor^13–18^. For applications that require very high sensitivity, high-quality factor resonators such as waveguide cavities are best suited^19^. On the other hand, planar resonators such as split ring resonators (SRRs) and defected ground structures (DGS) including complementary split ring resonators (CSRRs) and dumbbell-shape DGS resonators benefit from very low cost and compact size. As a result, they have been widely used in the fabrication of small sensors of moderate sensitivity^20–23^.

Because of these advantages, while waveguide cavities have been successfully employed for crack sensing^24–26^, considerably more research has been concentrated on the development of fracture sensors based on planar resonators^27–32^. For example, it has been demonstrated in^30^ that the presence of a crack on a metal surface can be easily detected by a shift in the resonance frequency of a CSRR. However, to our best knowledge, the theoretical foundations of sensing a crack in metallic surfaces have not been studied in depth. As a result, most studies in this field have been conducted based on optimization, where in some cases the research has gone in the wrong direction. For instance, considering that the frequency shift in a CSRR crack sensor was due to changes in the equivalent inductance of the CSRR, to improve the sensitivity, increasing the equivalent inductance was proposed in^31^. This was conducted by loading the sensing CSRR with a lumped inductance.

However, it was shown that this method leads in fact to much lower sensitivity^31^. On the other hand^32^, suggests that reducing the capacitance of the CSRR by removing slots from its metallic patch leads to higher sensitivity. However, as will be shown, theoretical analysis and EM simulations contradict this claim.

These observations prompted us to carry out theoretical studies on different aspects of sensing based on microwave resonators. Specifically, the main objectives of the article are as follows:

1. To perform a thorough analytical investigation of different planar configurations to achieve guidelines for the development of highly sensitive crack sensors based on different DGS resonators. Both the circuit model and the electromagnetic (EM) perspectives are used in this investigation.
2. To validate the key conclusions of the theoretical study both numerically and experimentally.
3. To use the outcomes of the study to develop a number of new, more sensitive metal fracture sensors.

Regarding the third goal mentioned above, two novel crack sensors with improved sensitivities based on distinct types of DGS resonators are being developed. Furthermore, a third, unique crack sensor based on folded dumbbell-shape DGS resonators is built to demonstrate the vast potential of DGS resonators. It is shown that, in contrast to existing crack sensors described in the literature, the proposed dumbbell-shape sensor can detect cracks of any orientation.

The equivalent circuit parameters of each of the three sensors placed over metal surfaces with and without a fracture are extracted and discussed to gain a better understanding. All three new sensors are fabricated and tested for their ability to detect cracks on a metal surface.

The remaining of this work is organized as follows: The theoretical foundations and sensitivity analysis of microwave resonators, especially for sensing cracks on a metal surface, are presented in Section “Microwave sensing: resonant elements and theoretical foundations”. The section includes the sensitivity analysis of different types of resonators from the circuit model and EM field perspectives. The insight obtained from the theoretical analysis is then used in Section “Crack detection using defected ground structures” to design various DGS resonators for crack sensing with different sensitivities. Discussion on the performance of the designed sensors based on the extraction of the equivalent circuit models, as well as the experimental validations are also presented in Section “Crack detection using defected ground structures”. Finally, the main conclusions are highlighted in Section “Conclusion”.

## Microwave sensing: resonant elements and theoretical foundations

This section aims to provide the principles and theoretical foundations for crack sensing, with a special emphasis on planar metallic resonators as well as planar complementary resonators such as DGS resonators. The materials presented in this section will be used in the next section to analyze and design crack sensors with improved characteristics.

**Figure 1:**
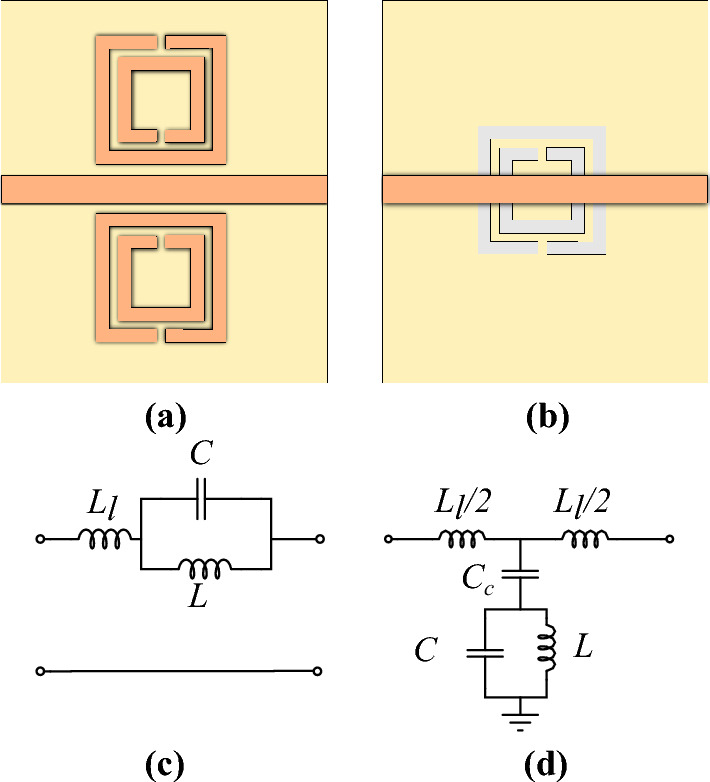
Illustrations of (**a**) an SRR-loaded and (**b**) a CSRR-loaded microstrip lines, and their equivalent circuit models (**c**) and (**d**), respectively.

### Planar resonant elements: split ring resonator (SRR) versus complementary split ring resonator (CSRR)

Selecting the best planar microwave resonator as the key component is the first step in designing a highly sensitive microwave sensor for crack detection. Considering compactness as one of the main figures of merit for different types of sensors, many studies in this field have been focused on the application of metamaterial-inspired resonators. At this point, we have usually two choices for planar ring-type structures: a metallic resonator (more precisely, an SRR) or its complementary counterpart (i.e., a CSRR). A crucial question is to determine which resonator, SRR or CSRR, leads to higher sensitivity. To address this question, behaviors of SRR-loaded and CSRR-loaded microstrip lines are studied in this subsection. Illustrations of both structures and corresponding equivalent circuits are presented in Fig. 1a–d. The models of Fig. 1c,d are well established in the literature and many quantitative analyses that support them have been provided (e.g., in^33^]).

To maintain consistency, symbols *L* and *C* without indices are used throughout the paper to refer to the inductance and capacitance of the resonant elements (SRR, CSRR, DGS, etc.), while other inductors and capacitors are distinguished by their indices. Note that for the circuit model of the SRR-loaded line, the pair of SRRs are magnetically coupled to the line. However, this magnetic coupling can be transformed to a circuit like the one shown in Fig. 1c, as pointed out in several sources, e.g., in^34^. Therefore, in the circuit model of Fig. 1c, which corresponds to the SRR-loaded transmission line (TL), *L* models the inductance of the metallic ring, while *C* represents the gap capacitor formed by the slit, and $L_\ell$ represents the inductance of the microstrip line. Similarly, in the circuit model of Fig. 1d, which corresponds to the CSRR-loaded line, $C_c$ is the coupling capacitance between the microstrip line and the CSRR, the capacitance *C* represents the electric coupling between the central metallic patch of the CSRR and the surrounding ground plane, and *L* models the inductor formed by the two half-rings as well as the short metallic strip that bridges from the CSRR metallic patch to the ground plane^33,35^.

It is clear from the equivalent circuit models that both SRR and CSRR provide a transmission zero (TZ) as well as a transmission pole. However, usually, the frequency of the TZ is used for sensing applications. It is important to note that the TZ for the SRR-loaded line occurs when the admittance of the parallel LC resonator vanishes; therefore the TZ is located at

1 $$\begin{aligned} f= 1/2 \pi \sqrt{L C}. \end{aligned}$$

In contrast, the TZ for the CSRR-loaded line occurs when the impedance (rather than admittance) of the shunt branch vanishes. Therefore, the TZ for the CSRR-loaded line is located at

2 $$\begin{aligned} f = 1/2 \pi \sqrt{L (C+C_c)}. \end{aligned}$$

Given the differences between an SRR and a CSRR, such as the difference in topology and thus equivalent inductance and capacitance, as well as the fact that the TZ in the SRR-loaded TL occurs at the resonance frequency of a parallel LC resonator, whereas the TZ in the CSRR-loaded TL occurs at the resonance frequency of a series LC resonator, the following questions arise:

1. The first question to consider is whether a parallel LC configuration is more sensitive to variations in *L* or *C* than a series LC resonator, or vice versa. Knowing the answer to this question can help determine which type of resonator is best suited for developing a highly sensitive sensor.
2. The second matter to investigate is whether the sensitivity of the resonance frequency (in either of the parallel or series LC configurations) to the variations of its capacitance or inductance can be improved by changing the ratio of *L* over *C*, or using larger or smaller *L* and *C* values. Answering this question is important, as it can help us optimize the sensitivity of a sensor by adjusting the values and the ratio of the equivalent inductance and capacitance of the utilized resonator.
3. Let us assume two resonators, with identical resonance frequency but different values of *L* and *C*, are used as the key components in two sensors based on variations of inductance. The question is whether the resonator with larger inductance leads to higher sensitivity, or if the opposite is true. For instance, while the resonance frequency of an SRR is almost equal to that of a CSRR with identical complementary layout, their *L* and *C* values are quite different. The question is whether using the SRR, which typically has a larger inductance, results in higher sensitivity in a sensor based on the variations of inductance. In contrast, an SRR has a smaller capacitance than a CSRR with an identical complementary layout. Therefore, if sensing is conducted based on the variations of the equivalent capacitance, we must ask whether the CSRR-based sensor is more sensitive than the SRR-based sensor.
4. Specifically in the case of a CSRR, which mechanism for crack sensing leads to higher sensitivity; variations in the CSRR inductance or capacitance?
5. And finally, a relevant question to explore is whether increasing the inductance or capacitance of a CSRR by loading it with a lumped inductor or capacitor may improve its sensitivity.

Answers to these questions from the circuit model and electromagnetic perspectives will be sought in the next subsections.

### Sensitivity analysis: equivalent circuit model perspective

**Figure 2:**
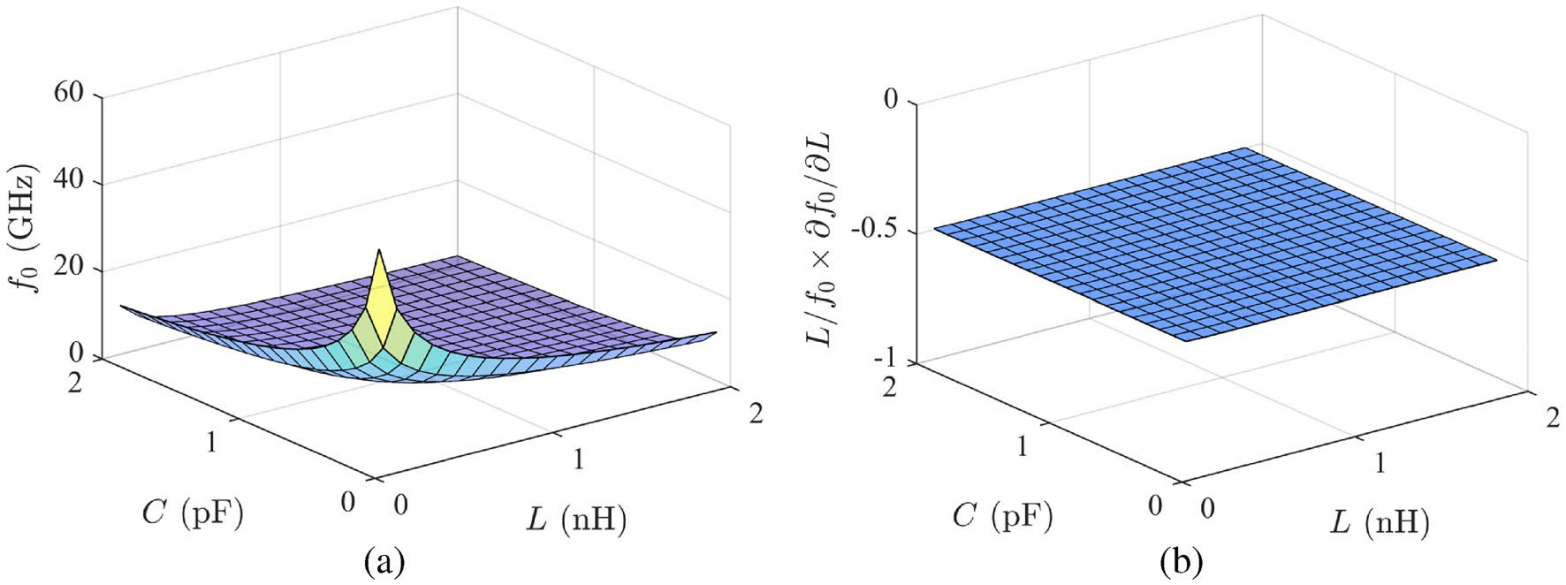
Three-dimensional plots of (**a**) the resonance frequency of an LC resonator as a function of its components *L* and *C*, and (**b**) the sensitivity of an LC resonator as defined in (7), i.e., the normalized derivative of $f_0$, for one percent increase in *L* for different values of *L* and *C*.

To address the questions raised, this subsection will focus on conducting a sensitivity analysis of various types of LC resonators, which serve as the primary components of sensors. This will be done through an examination of the equivalent circuit model. For convenience, we start by assuming a parallel LC resonator as the main building block of a sensor. We also assume that the output variable of the sensor is the resonance frequency of the parallel LC resonator, i.e.,

3 $$\begin{aligned} f_0 = 1/2 \pi \sqrt{L C} \end{aligned}$$

that varies due to changes in the inductance *L* of the resonator. With these assumptions, the rate of changes in the resonance frequency $f_0$ to changes in *L* (or simply $\partial f_0 / \partial L$) may be considered as the sensitivity of the sensor. From (3), we obtain

4 $$\begin{aligned} \partial f_0 / \partial L = -\frac{1}{4\,\pi \,{\sqrt{C\,L^3}}}, \end{aligned}$$

which shows that the derivative of $f_0$ with respect to *L* may be increased by using smaller values of *L* and *C*. This is also clear from the three-dimensional plot of $f_0$ as a function of *L* and *C*, which is shown in Fig. 2a. So, it may be concluded that the sensitivity of a sensor based on the variations of the inductance of a parallel LC resonator can be increased by using smaller *L* and/or *C*.

However, it is important to note that the resonance frequency is also the operating frequency of the sensor. Considering the higher cost of the realization and complexity of the measurement at higher frequencies, the normalized value (rather than the absolute value) of the frequency shift is used as a figure of merit for comparing the sensitivity of sensors operating at different frequencies. In other words, as shown in^36^, the sensitivity of a dependent parameter *y* to an independent parameter *x* is defined as the ratio of the normalized variation of the dependent parameter $\Delta y/y$ to the normalized variation of the independent parameter $\Delta x /x$, that is

5 $$\begin{aligned} S^y_x \approx \frac{\Delta y /y}{\Delta x /x}. \end{aligned}$$

On that basis, the first-order classical sensitivity, also known as the Bode sensitivity, is defined as^36^

6 $$\begin{aligned} S^y_x = \frac{x}{y}\frac{\partial y}{\partial x}. \end{aligned}$$

Using this definition, the sensitivity of the measure parameter, i.e., the resonance frequency of a parallel LC resonator, to the variations of its inductance is found to be

7 $$\begin{aligned} S^{f_0}_L = \frac{L}{f_0}\frac{\partial f_0}{\partial L} = -\frac{1}{2}, \end{aligned}$$

which tells that the normalized shift of the resonance frequency due to a specific normalized change in the inductance is a constant independent of the original values of *L* and *C*. The negative sign indicates that an increase in *L* results in a decrease in the resonance frequency. In simple terms, the equation states that regardless of the ratio of *L* to *C* and even regardless of the operating frequency, a 1% increase in the inductance *L* of the resonator results in a 0.5% decrease in its resonance frequency. Based on this discussion, the Bode sensitivity is used in the rest of the article (even though not explicitly stated). The Bode sensitivity of a parallel LC resonator as defined in (7), i.e., the normalized derivative of $f_0$, for one percent increase in *L* for different values of *L* and *C* is shown in Fig. 2b. The figure clearly confirms that the sensitivity of the sensor is independent of the values of *L* and *C*, and consequently independent of the operating frequency.

Note that considering the symmetry of Eq. (3), it can be inferred that the sensitivity of the resonance frequency of a parallel LC resonator to its capacitance is also $-1/2$, that is a constant independent of the values of *L*, *C*, and the operating frequency. Similar conclusions can be made for a series LC resonator since the equation for the resonance frequency of a series LC resonator is identical to that of a parallel LC resonator.

To answer the third question, let us move one step further and analyze the Bode sensitivity of an LC resonator to the ultimate input variable of the sensor. For this purpose, without loss of generality, a sensor for sensing the permittivity of a dielectric is considered. Therefore, the sensor can be composed of an LC resonator whose capacitive part is loaded with a small sample of the material under test. The output parameter is the shift in the resonance frequency of the resonator due to the variations of capacitance *C*. Therefore, the sensitivity of the sensor to the ultimate input variable, i.e., the permittivity of the loading material is

8 $$\begin{aligned} S^{f_0}_{\varepsilon _r} = S^{f_0}_{C} \cdot S^{C}_{\varepsilon _r} = -\frac{1}{2} \frac{\varepsilon _r}{C}\frac{\partial C}{\partial \varepsilon _r} \approx -\frac{1}{2} \frac{\Delta C /C}{\Delta \varepsilon _r /\varepsilon _r}. \end{aligned}$$

The equation simply states that the sensitivity of the sensor can be improved if the normalized variation of the capacitance due to a specific variation in the permittivity is enhanced. In a permittivity sensor, this can be achieved by loading the whole effective volume of the capacitor with the dielectric under test. Since in most permittivity measurement applications such as microfluidic sensors, utilizing a very small amount of material under test is desired, it can be concluded that using small capacitors with confined electric field results in higher sensitivity.

With reference to the third question, assume two resonators with identical resonance frequencies but different values of *L* and *C* are used as two permittivity sensors based on variations in the capacitance *C*. It can be counter-intuitively concluded that the resonator with smaller capacitance leads to higher sensitivity. For instance, an SRR may provide a higher sensitivity for permittivity measurement compared to a CSRR with the same layout, simply because the SRR capacitor is small and confined to a limited area whereas that of the CSRR is larger and distributed around the four edges of its metallic patch.

Rewriting Eq. (8) for a sensor based on the variations of inductance that is used for sensing the permeability of different materials gives

9 $$\begin{aligned} S^{f_0}_{\mu _r} = S^{f_0}_{L} \cdot S^{L}_{\mu _r} = -\frac{1}{2} \frac{\mu _r}{L}\frac{\partial L}{\partial \mu _r} \approx -\frac{1}{2} \frac{\Delta L /L}{\Delta \mu _r /\mu _r}. \end{aligned}$$

It is inferred from this equation that the sensitivity of the sensor can be improved if the normalized variation of the inductance due to a specific variation in the permeability is enhanced. This can be achieved if the inductive section of the sensor is confined to a small volume. On that bases, a CSRR, with a small confined inductive area around its bridge section may be a more suitable resonator for sensing the permeability of a small sample under test.

The behavior of a crack sensor can be explained in a similar manner. It can be shown that the inductance *L* of a resonator over a crack in a metal surface is a function of the crack volume ${\textrm{V}}$. Therefore, the sensitivity of such a resonator can be expressed as

10 $$\begin{aligned} S^{f_{0}}_{\textrm{V}} = S^{f_0}_{L} \cdot S^{L}_{\textrm{V}} = -\frac{1}{2} \frac{{\textrm{V}}}{L}\frac{\partial L}{\partial {\textrm{V}}} \approx -\frac{1}{2} \frac{\Delta L /L}{\Delta {\textrm{V}} /{\textrm{V}}}. \end{aligned}$$

The equation shows that for a specific variation in the crack volume, the Bode sensitivity of the resonator depends on the normalized variations of its inductance, i.e., $\Delta L/L$. Therefore, a higher sensitivity is achieved by using a resonator with a small inductance *L* that is confined to a limited volume. It will be shown in the next section that this can be achieved in DGS resonators such as CSRRs.

In summary, answers to the questions raised at the beginning of this section, which are the guidelines for the design of high-sensitivity sensors, are as follows:

1. The sensitivity of a sensor operating based on the shift of the resonance frequency may not be improved simply by changing the core component of the sensor from a parallel to a series LC resonator, or vice versa.
2. Considering the normalized frequency shift as the output variable, the sensitivity of the sensor (either based on a parallel or a series LC resonator) is independent of the absolute values and the ratio of *L* and *C*.
3. Although somewhat counter-intuitive, a resonator with a small capacitive section is more suitable for sensing based on the changes in the capacitance; for instance, sensing the permittivity of an unknown material. Also, a resonator with a small inductive section is best for sensing based on the changes in the inductance (for instance sensing the permeability of a material or cracks on a metal surface). In other words, generally a crack sensor based on a CSRR may have higher sensitivity compared to a sensor based on an SRR with the same complementary layout.
4. Generally, the capacitance of a CSRR is relatively large and distributed. Therefore, in a well-designed CSRR with a small confined inductive section, sensing based on the variations of inductance leads to higher sensitivity compared to sensing based on the variations in the capacitance. This simply means a CSRR is more suitable for sensing based on the variations of inductance; for instance for sensing permeability or sensing a crack in a metallic surface. In contrast, an SRR is best suited for sensing based on the variations of capacitance, for instance for sensing the permittivity or a crack in a dielectric.
5. Finally, Eqs. (8)–(10) show that the sensitivity of the sensors is proportional to $\Delta L/L$ or $\Delta C/C$. Therefore, increasing the inductance or capacitance of a resonator by loading it with a lumped inductor or capacitor cannot improve its sensitivity simply because for a lumped component $\Delta L/L = \Delta C/C = 0$.

### Sensitivity analysis: electromagnetic field perspective

Some of the conclusions of the previous section can be further clarified from the electromagnetic field point of view. We remind that considering an arbitrary resonator, the aim is to investigate which mechanism leads to a higher sensitivity to a crack on a metal surface; changes in the inductance (or equivalently magnetic field), changes in the capacitance (or equivalently electric field), or even changes in both *L* and *C* (or ${\textbf {H}}$ and ${\textbf {E}}$).

Let us consider a cavity resonator that one of its sidewalls is replaced with the metal surface under test. Assuming that the resonance frequency of the cavity with faultless surfaces occurs at $f_0$, a crack in the metal surface under test results in a shift $\Delta f_0$ in the resonance frequency. The normalized frequency shift can be calculated by^37^

11 $$\begin{aligned} \frac{\Delta f _0}{f_0}=\frac{{f_0' }-{f_0 }}{f_0}=- \frac{\Delta {W}_{m}-\Delta {W}_{e}}{{W}_{m}+{W}_{e}} \end{aligned}$$

In this equation, $f_0$ and $f_0'$ are the resonance frequencies of the cavity before and after perturbation by the crack, $W_m$ and $W_e$ denote the total magnetic and electric energies stored in the whole volume of the cavity, and $\Delta W_m$ and $\Delta W_e$ are the differences in the stored magnetic and electric energies due to the perturbation of the cavity. Assuming the perturbation is small enough, which is true in the case of targeted cracks, the total stored energy $W_m + W_e$ can be approximated by the sum of the stored magnetic and electric energies in the unperturbed cavity. Also, $\Delta W_m$ and $\Delta W_e$ can be approximated with the magnetic and electric energies stored in the volume of the crack. Substituting these values in (11) gives

12 $$\begin{aligned} \frac{\Delta f _0}{f_0} \approx - \frac{{\int _{\textrm{crack}} }( \mu {\left| {H}_{0}\right| }^{2}- \epsilon {\left| {E}_{0}\right| }^{2})dv}{{\int _{\textrm{cavity}} }( \mu {\left| {H}_{0}\right| }^{2} + \epsilon {\left| {E}_{0}\right| }^{2})dv}. \end{aligned}$$

This relation gives valuable insight into the behavior of the sensor. In short,

1. The numerator of the relation clearly shows that the frequency shift due to the electric and magnetic stored energies in the volume of the crack are in opposite directions. In other words, the equation shows that increasing the stored magnetic field in the cavity (for instance by introducing a hole or crack in the vertical walls of the cavity) reduces the resonance frequency. In contrast, increasing the stored electric field in the cavity (for instance by introducing a hole in the center of top or bottom surfaces of the cavity) increases the resonance frequency.
2. While based on the relation $f_0 = \ 1/ 2\pi \sqrt{L C}$ one may expect to achieve a higher frequency shift if both *L* and *C* are increased (or decreased), Eq. (12) shows that generally, stored electric and magnetic energies in a perturbation such as a crack have destructive effects.
3. Based on the previous points, generally if a crack equally perturbs both the electric and the magnetic fields of the cavity, their effects on the frequency shift cancel each other, and no frequency shift will be observed. Therefore, one method for improving the sensitivity (i.e., achieving a larger frequency shift) is to perturb the cavity such that the variation of one of the stored energies becomes negligible (i.e., either $\Delta W_e \approx 0$ or $\Delta W_m \approx 0$), while the other one is a strong function of the perturbation.

**Figure 3:**
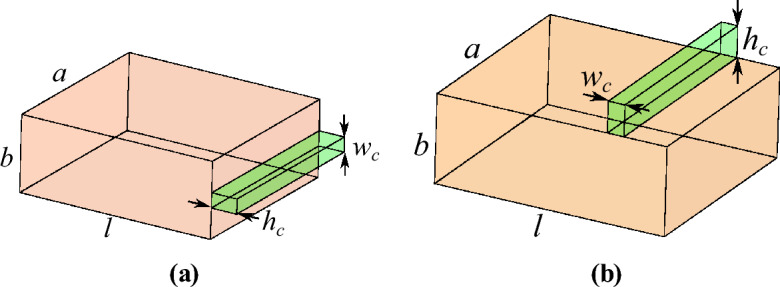
Illustrations of two configurations for sensing a crack on a metal surface using a half-wavelength cavity resonator when (**a**) the short-end of the cavity is replaced with the metal surface under test, and (**b**) the upper conductive surface of the cavity is replaced with the metal surface under test. The dimensions of the simulated cavity are $a = 10.67$ mm, $b = 4.32$ mm, and $l = 7.71$ mm. The crack has a width $w_c = 0.2$ mm, depth $h_c = 1.5$ mm, and length $l_c = a = 10.67$ mm.

To demonstrate this conclusion, different configurations of a rectangular cavity for sensing a crack are studied. The aim is to highlight that high sensitivity to a crack may be achieved if a proper sidewall of the rectangular cavity is replaced with the metal surface under test. However, as stated earlier, the concept is general and can be applied to other resonators including planar resonators. With this aim, two possible configurations for sensing a crack on a metal surface using a half-wavelength cavity resonator are illustrated in Fig. 3a,b. In the first configuration, the short end of the cavity is replaced with the metal surface under test, whereas, in the second configuration, the upper conductive surface of the cavity is replaced with the metal surface under test. Note that due to the PEC boundary conditions, the electric field vanishes at the sidewalls of the cavity, while the magnetic field takes its maximum value along the sidewalls. Therefore, it can be concluded that a crack in the end wall of the cavity where the electric field is negligible, and the magnetic field is quite strong, leads to a pronounced downward frequency shift. In contrast, for a crack in the top side of the cavity $\Delta W_e \approx \Delta W_m$. Therefore, based on (11) the frequency shift $\Delta f_0$ is nearly zero.

To validate this conclusion, a rectangular waveguide cavity with the resonance frequency $f_0 = 24$ GHz is simulated, and its electric and magnetic fields within the volume of the cavity are computed using the full-wave High-Frequency Structure Simulator (HFSS). The simulation is repeated after perturbing the cavity by introducing a crack (1) to the terminating wall, and (2) to the top surface of the cavity. The dimensions of the cavity and crack are listed in the caption of Fig. 3. The computed stored electric and magnetic energies in the unperturbed cavity, as well as the stored energies in the volume of the crack in both cases, are listed in Table 1. The table also shows the frequency shift obtained by substituting the computed stored energies into Eq. (12). Note that as expected, when the crack is in the terminating wall of the cavity $\Delta W_e$ is much smaller than $\Delta W_m$. As a result, a relatively high frequency shift of 210 MHz is achieved. In contrast, almost no frequency shift is observed when the crack is in the top surface where $\Delta W_e \approx \Delta W_m$. For comparison, the resonance frequency of the structure, i.e., the frequency at which the imaginary part of the input impedance vanishes, is also computed using full-wave EM simulation, and listed in the table. Good agreement between computed resonance frequencies is observed. Slight differences are due to the approximations used in the calculation of the stored energies.

**Table 1:** Computed stored electric and magnetic energies in the unperturbed cavity, as well as the stored energies in the volume of the crack are listed. The table also compares the expected frequency shifts based on Eq. (12) to those obtained from full-wave EM simulations..

Structure	$W_e$	$W_m$	$\Delta W_e$	$\Delta W_m$	$f_0$ (GHz)	$f_0$ (GHz)
	(a.u)	(a.u)	(a.u)	(a.u)	(Eq. 12)	(EM simulated)
Without crack	6.33	6.33	0	0	24	24
Figure 3a	6.37	6.35	0.03	0.16	23.76	23.79
Figure 3b	6.306	6.30	0.0035	0.0016	24.004	24.008

Another important point to note is that by definition, at resonance, the stored electric energy in a resonator equals the stored magnetic energy, i.e., $W_e = W_m$. Therefore, assuming that the cavity is perturbed by a crack in the terminating wall where the electric field is negligible, hence $\Delta W_e \approx 0$, relation (11) can be reduced to

13 $$\begin{aligned} \frac{\Delta f_{0} }{f_{0}}=\frac{{f_{0'} }-{f_0 }}{f_0}= -\frac{1}{2}\frac{\Delta {W}_{m}}{ {W}_{m}}. \end{aligned}$$

The equation simply shows that the frequency shift does not depend on the absolute values of the stored electric and magnetic fields, but it depends on the relative change in the stored energy after getting perturbed. This is quite identical to the conclusions made in the previous subsection using equivalent circuit models.

As mentioned earlier, the presented analysis is general, and hence valid for any resonator with arbitrary geometry. Thus, the analysis can be also applied to planar resonators including CSRR and other DGS resonators if the boundaries of the resonators are assumed large enough.

The sensitivity analysis presented in the previous subsection, and the insight into the behavior of a resonator from the electromagnetic field perspective achieved in this subsection are the essential means that will be used in the next section to analyze and design sensitive crack sensors based on defected ground resonators. We will also use our findings to demonstrate that some of the crack sensors proposed in the literature have suboptimal sensitivity.

## Crack detection using defected ground structures

This section is devoted to the application of the presented theory for the development of several novel crack sensors. Specifically, the next two subsections are focused on the design of crack sensors with high sensitivity to cracks in a specific orientation. The third subsection aims at designing a novel crack sensor for detecting cracks with arbitrary orientations.

### A sensitive crack sensor based on a CSRR

Application of conventional double-ring CSRRs for crack sensing has been presented in^30^. The presented sensor in^30^ suffers from relatively low sensitivity. It has been claimed in^32^ that reducing the equivalent capacitance of the CSRR by etching out patterned strips from its metallic patch can improve the sensitivity of such crack sensor.

However, this is inconsistent with Eq. (10) and our conclusion in the previous section that states the sensitivity of a sensor based on the variations of the inductance is independent of its capacitance *C*. This will be also verified in this section thorough EM simulation of the structure. In addition to clarifying this issue, it is demonstrated in this section that the sensitivity of a crack sensor based on CSRRs can be dramatically increased simply by using a single-ring CSRR rather than a double-ring one. In other words, it will be shown that in contrast to what is claimed, the sensitivity improvement achieved in^32^ is due to the smaller equivalent inductance in the single-ring CSRR (and not due to the reduced capacitance). This is consistent with Eq. (10) and the fourth theoretical conclusion in Section “Sensitivity analysis: equivalent circuit model perspective” that states the sensitivity of a sensor based on the variations of inductance can be improved by using a resonator with small confined inductance. Prior to embarking on numerical analysis, it’s important to recall that the inductance of a strip positioned above a metallic surface can be determined by integrating the normal magnetic flux density across surface S. This surface lies between the strip and the return current path on the metallic surface. Hence, the resulting calculated equivalent inductance depends on factors such as the strip’s width, length, and its distance from the return current on the metallic surface. It is now evident that the presence of a crack in the metallic surface induces an augmented separation between the strip and its corresponding return current path on the metallic surface. As a result, an increase in the equivalent inductance of the strip over the cracked metallic surface is observed.

**Figure 4:**
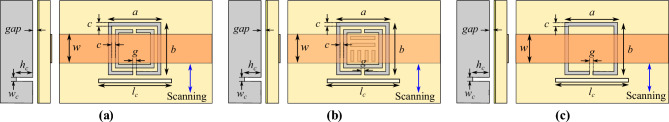
Top and side views of (**a**) the proposed crack sensor in^30^ based on a double-ring CSRR, (**b**) a crack sensor based on a double-ring CSRR, which is modified according to the method presented in^32^, and (**c**) the proposed high sensitivity crack sensor based on a single-ring CSRR. The width of the microstrip line is $w = 1.7$ mm. The dimensions of the CSRRs etched in the ground plane are $a = 3$ mm, $b=3$ mm, $g= 0.2$ mm, and $c=0.2$ mm. The crack to be sensed, which has a width $w_c=0.2$ mm, length $l_c=4$ mm, and depth $h_c=2$ mm, is devised on an Aluminum block. The metallic block and the ground plane are separated by an air gap $gap = 0.2$ mm.

In light of this conceptual framework, the subsequent sections undertake a comparison of two crack sensors based on double-ring CSRRs with and without etched strips. These results are further compared with the sensitivity of a sensor employing a single-ring CSRR. As depicted in Fig. 4a through 4c, the three sensors share similarities in that each comprises a 50 $\Omega$ microstrip line loaded with a CSRR. The illustrations also highlight that the sensing structures are positioned above the metal surface being tested, with an intervening air gap separating them.

To compare the sensitivities, the three sensors are simulated using HFSS at two states: (1) when the sensor is placed over a faultless metal surface, and (2) when the sensor is placed over a cracked metal surface. For simulation purposes, the parameters of the Isola *I-TeraMT (R)* substrate with a thickness $h= 0.762$ mm, a relative permittivity $\epsilon _r=3.45$, and a dielectric loss tangent $\tan (\delta ) = 0.0031$ are used. Other dimensions of the structures as denoted in Fig. 4a–c are as follows: the width of the microstrip line is $w = 1.7$ mm, which corresponds to a 50 $\Omega$ characteristic impedance, and the dimensions of the CSRRs etched in the ground plane are $a = 3$ mm, $b=3$ mm, $g= 0.2$ mm, and $c=0.2$ mm. The crack to be sensed, which has a width $w_c=0.2$ mm, length $l_c=4$ mm, and depth $h_c=2$ mm, is devised on an Aluminum block. The metallic block and the ground plane are separated by an air gap $gap = 0.2$ mm. The three CSRRs have identical outside dimensions; however, the inner etched ring in the CSRR of Fig. 4a is removed to form the single-ring CSRR in Fig. 4c. The strips etched out from the CSRR of Fig. 4b are 0.2 mm wide and 0.2 mm apart. For EM simulation purposes, the microstrip line is fed through SMA connectors, and the whole structure is enclosed by a cubic vacuum with radiation boundary conditions applied to its surfaces. “Mesh Settings” are crucial parameters that require specific attention, as there exists a substantial likelihood that the automated meshing feature in commercial electromagnetic (EM) simulator packages might not allocate adequately fine meshes to the interior of the crack. Consequently, it becomes necessary to manually designate dense meshes for the volume within the crack.

**Figure 5:**
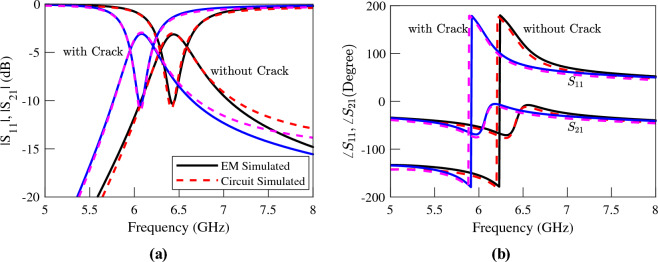
Simulated (**a**) magnitude and (**b**) phase of the transmission and reflection coefficients of the sensor of Fig. 4a, when the sensor is located above an Aluminum block with and without a crack.

**Figure 6:**
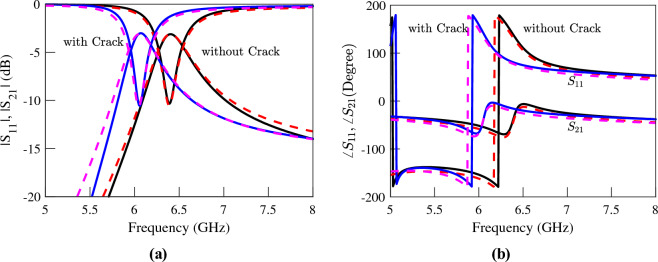
Simulated (**a**) magnitude and (**b**) phase of the transmission and reflection coefficients of the sensor of Fig. 4b, when the sensor is located above an Aluminum block with and without a crack.

**Figure 7:**
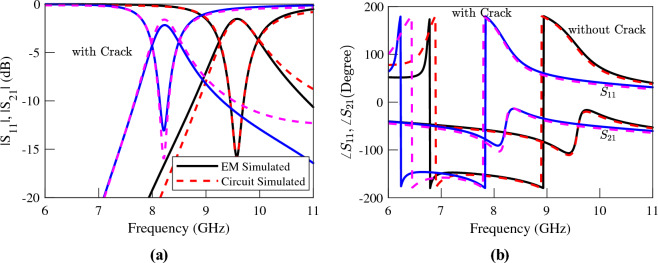
Simulated (**a**) magnitude and (**b**) phase of the transmission and reflection coefficients of the sensor of Fig. 4c when the sensor is located above an Aluminum block with and without a crack.

The EM simulated transmission and reflection coefficients of the sensors above an Aluminum block with and without a crack are plotted (using solid lines) in Figs. 5, 6 and 7. The resonance frequency of each CSRR over the faultless metal surface is considered as the reference for the calculation of the sensitivity of the corresponding sensor. The EM simulations in Fig. 5 show a 350 MHz shift in the resonance frequency of the first sensor due to the crack in the faulty metal. This shift corresponds to a normalized sensitivity of $5\%$. As shown in the simulation results presented in Fig. 6 the sensor based on the double-ring CSRR with etched strips has almost identical resonance frequencies, and hence the same sensitivity to the crack. Note that this is contrary to what is claimed in^32^, and is consistent with our second conclusion in Section “Sensitivity analysis: equivalent circuit model perspective” that the sensitivity of a sensor based on the variations of inductance cannot be improved simply by reducing its capacitance or increasing the ratio of *L*/*C*. In contrast, the frequency shift of the single-ring CSRR due to the presence of a crack in the metal block is about 1350 MHz, which is equivalent to $14\%$ frequency shift. This is almost three times higher than the frequency shift in the sensors based on double-ring CSRRs. Again, this result is supported by the fourth conclusion in Section “2.2”, which states that the sensitivity of a sensor based on the variations of inductance can be improved if a resonator with a small confined inductive part is used. Note that the inductive section of the double-ring CSRR includes the metallic bridge as well as the two metallic half-rings. In contrast, the inductive section of the single-ring CSRR is formed only by the metallic bridge, which is confined to a small area.

Equivalent circuit models of the sensors may help to better understand the effect of a crack on the resonance frequency of the sensing CSRRs and to explain the reason behind the higher sensitivity of the sensor with a single-ring CSRR. A relatively detailed equivalent circuit model for a CSRR over a metal surface with and without a crack has been proposed in^32^. However, due to too much detail, the model does not give much insight into the physics of the problem. A simpler equivalent circuit model that also includes the cross-coupling of the CSRR to both electric and magnetic fields of the loaded line has been proposed and utilized in^33,38^. However, since the symmetry planes of the CSRRs considered in this study are orthogonal to the loaded TLs, assuming a pure electric coupling between the CSRR and the loaded TL would be sufficient^33,35,38^. As a result, as will be demonstrated in the following, the basic equivalent circuit presented in Fig. 1d can accurately model both sensors based on single- and double-ring CSRRs, and it is very useful in understanding the physics behind different sensitivities of the presented sensors. We remind that in this model, *L* and *C* respectively model the inductance and capacitance of the CSRR itself, the capacitance $C_c$ represents the electric coupling between the microstrip line and the CSRR, and $L_\ell$ models the inductance of the piece of TL above the CSRR.

The procedure presented in^39^ is used to extract the values of the circuit model elements. The extracted parameters for the sensors at both states, i.e., over the faultless and over the cracked metal surfaces, are listed in Tables 2, 3 and 4. For comparison, the circuit simulated scattering parameters using the extracted parameters of each sensor are also presented (using dashed lines) next to the corresponding EM simulated graphs in Figs. 5, 6 and 7. In order to consider the effect of losses, a small resistor ranging between $0.1~\Omega$ and $0.15~\Omega$ in series to the equivalent inductor is included in the equivalent circuits. The good agreement between the circuit and EM simulation results confirms both the circuit model and the utilized parameter extraction method. The phase of the circuit and the EM-simulated scattering parameters are also in good agreement. However, for conciseness, the graphs of phases are not shown.

**Table 2:** The extracted parameters for the sensor of Fig. 4a at two states; over the faultless and over the cracked metal surface..

Parameter	Over faultless metal	Over cracked metal	%
*L* (nH)	0.29	0.33	16.26
*C* (pF)	1.86	1.79	− 4.17
$L_\ell$ (nH)	0.93	0.95	1.42
$C_c$ (pF)	0.31	0.30	− 2.12

**Table 3:** The extracted parameters for the sensor of Fig. 4b at two states; over the faultless and over the cracked metal surface..

Parameter	Over faultless metal	Over cracked metal	%
*L* (nH)	0.32	0.39	23
*C* (pF)	1.68	1.51	− 10
$L_\ell$ (nH)	0.92	0.922	1.42
$C_c$ (pF)	0.28	0.26	− 7.28

**Table 4:** The extracted parameters for the sensor of Fig. 4c at two states; over the faultless and over the cracked metal surface..

Parameter	Over faultless metal	Over cracked metal	%
*L* (nH)	0.11	0.16	46.63
*C* (pF)	2.18	2.00	− 8.02
$L_\ell$ (nH)	1.01	0.99	− 1.73
$C_c$ (pF)	0.31	0.30	− 2.88

The circuit models and the extracted parameters may be used to justify the higher sensitivity of the proposed sensor based on a single-ring CSRR. Let us start by comparing the extracted parameters of the three sensors when they are sensing a faultless metal surface. Comparing Tables 2, 3 and 4, reveals that the values of $L_\ell$, $C_c$, and *C* for the three sensors over faultless metal are almost equal. (The values of the capacitors $C_c$ and *C* for the CSRR with etched strips are slightly smaller.) However, the value of *L* for the double-ring CSRRs with and without etched strips are respectively $L=0.32$ nH and $L=0.29$ nH, which are almost three times that of the single-ring CSRR with $L =0.11$ nH. Of course, this is expected as *L* in the double-ring CSRRs models the two half-ring strips as well as the short bridge that connects the CSRR to the ground plane, whereas, the inductance *L* in the single-ring CSRR is only formed by the short bridge.

Now let us compare the extracted parameters when the sensors are sensing the crack in the faulty metal surface. As listed in Tables 2 and 3, the normalized increase in the inductance *L* for the CSRR with etched strips is $\Delta L/L = 23\%$, which is slightly larger than that of the CSRR without the etched strip with $\Delta L/L = 16\%$. However, since the crack also causes a larger decrease in the capacitance values *C* and $C_c$ of the double-ring CSRR with etched strips, the overall frequency shifts in the two sensors are identical. In contrast, the normalized change in the equivalent inductance of the single-ring CSRR is $\Delta L/L = 46\%$. Note that the absolute change of the inductance between the two states, i.e., $\Delta L$, is almost equal ($\Delta L$ $\approx$ 0.05 nH) for the three sensors. However, as demonstrated by Eq. (10), the factor that determines the sensitivity of the sensor is the normalized variations of the inductance, i.e., $\Delta L/L$. This factor for the sensor based on a single-ring CSRR is three times that of the sensors based on a double-ring CSRR. This justifies the three times higher sensitivity of the proposed single-ring CSRR sensor.

The higher sensitivity of the proposed sensor also can be explained using the EM field analysis presented in the previous section. Consider the double-ring CSRR whose magnetic field is produced not only by the bridge strip but also by the two half-ring metallic strips. Therefore, its magnetic field is distributed almost in the whole volume around the CSRR. In contrast, the magnetic field of the single-ring CSRR is confined to a small volume around the bridge. As a result, as shown by Eq. (13) the normalized changes of the stored magnetic field due to the presence of the crack in the single-ring CSRR is larger, and consequently, the frequency shift is more pronounced. Also note that as predicted by (13), since the mechanism of sensing is based on the variations of the stored magnetic field a negative shift in the resonance frequency is observed.

**Figure 8:**
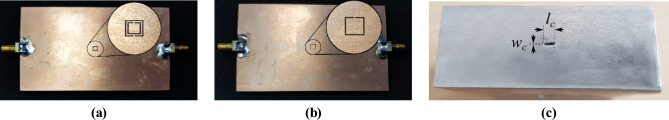
Photographs of the fabricated prototype of (**a**) a crack sensor based on a double-ring CSRR, (**b**) the proposed crack sensor based on a single-ring CSRR, and (**c**) the aluminum block under test with a crack.

**Figure 9:**
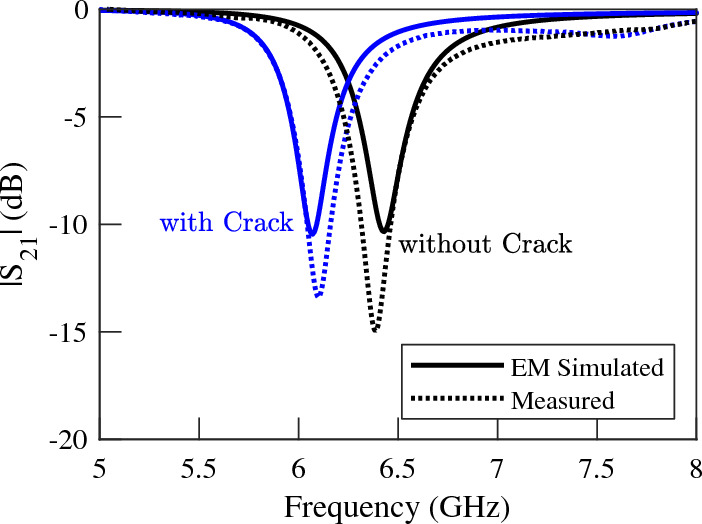
Measured (dotted lines) and EM simulated (solid lines) magnitude of the transmission coefficients of the sensor of Fig. 8a when the sensor is located above an Aluminum block with and without a crack. A $5\%$ frequency shift is observed.

**Figure 10:**
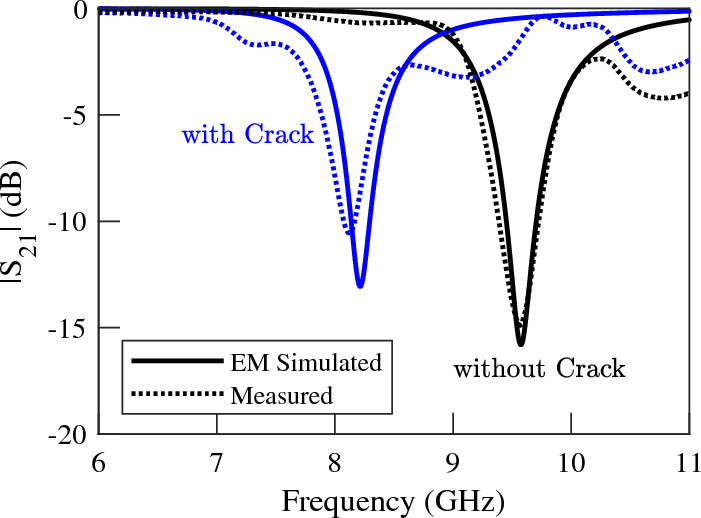
Measured (dotted lines) and EM simulated (solid lines) magnitude of the transmission coefficients of the sensor of Fig. 8b when the sensor is located above an Aluminum block with and without a crack. A $14\%$ frequency shift is observed.

In order to experimentally validate the presented concept and numerical results, prototypes of crack sensors of Fig. 4a,c are fabricated and their scattering parameters while sensing faultless and faulty metal surfaces are measured. Photographs of the fabricated prototypes are presented in Fig. 8a,b. Also, a photograph of the metallic surface under test, which is an aluminum block with a crack is shown in Fig. 8c. All the dimensions and the utilized materials of the prototypes correspond to the simulated structures. To avoid cluttered graphs, the measured transmission coefficients of the two sensors are plotted in Figs. 9 and 10. However, for easier comparison, the EM-simulated results are also presented next to the measured ones. As shown in the figures, the measured results are in good agreement with the computed ones. The experimental results confirm a $5\%$ shift in the resonance frequency of the first sensor due to the presence of a crack in the metal surface under test. The measured frequency shift observed in the second sensor is about $14\%$, which is identical to the computed value. In short, the presented concept and analytical results are validated by the good agreement between the numerical and experimental data.

Note that small fluctuations at high frequencies, which are observed in the measurement curves in Fig. 10, are due to the step discontinuity between the SMA connectors and the microstrip line. This effect can be mitigated by tapering the two ends of the microstrip line. It is also worth noting that when monitoring metal surfaces with limited width, for instance, railways, SMA connectors do not cause any issue because the connectors will be located on the two sides of the metallic surface. On other surfaces, however, other types of connectors or feeding scenarios can be used. For instance, the TL can be fed by a pair of through-hole straight SMA connectors mounted on the top side of the substrate.

### Sensitivity improvement using folded dumbbell-shape DGS resonators

It was shown in the previous subsection that a single-ring CSRR with a confined magnetic field can be used to achieve a crack sensor with high sensitivity. This subsection aims to show that guidelines extracted from the theoretical analysis can be used to develop other types of defected ground structures such as folded dumbbell-shape DGS resonators to achieve even higher sensitivity.

**Figure 11:**
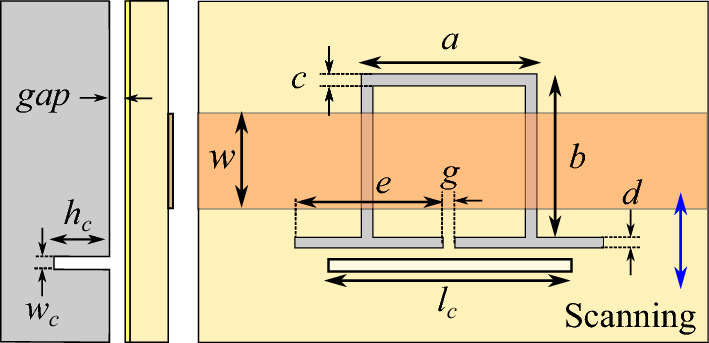
Illustrations of the top and side views of the proposed sensor based on a folded dumbbell-shape DGS located above an Aluminum block for crack sensing.

**Figure 12:**
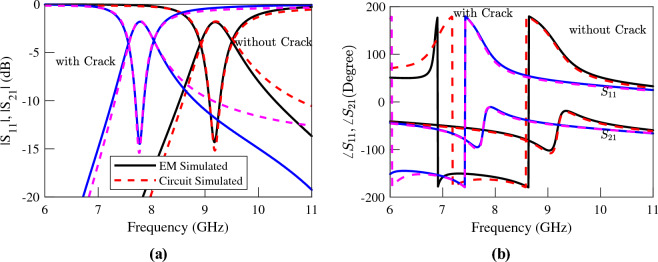
Simulated (**a**) magnitude and (**b**) phase of the transmission and reflection coefficients of the sensor of Fig. 11 when the sensor is located above an Aluminum block with and without a crack. A $15.5\%$ frequency shift is observed.

An illustration of the proposed sensor is shown in Fig. 11. The sensor is composed of a microstrip line loaded with a folded dumbbell-shape DGS. Comparing the layout of the utilized DGS resonator to the CSRR resonators of the previous section reveals that while still the inductive section of the resonator is limited to the bridge strip, the dimensions of the rectangular defects, i.e. *e* and *d*, give a higher freedom to adjust the sensitivity of the sensor to different cracks. For instance, from the theoretical conclusions of the previous section we know that the sensitivity of a crack sensor can be improved if the variations of the normalized equivalent inductance, i.e., $\Delta L/L$ due to a crack in the metal surface under test is enhanced. This can be achieved if a DGS resonator with quite narrow and long rectangular defected areas is used. For demonstration, in this section, the performance of a crack sensor based on such folded dumbbell-shape DGS is studied. Similar to the previous sensors, the parameters of the Isola *I-TeraMT (R)* substrate with a thickness $h= 0.762$ mm, a relative permittivity $\epsilon _r=3.45$, and a dielectric loss tangent $\tan (\delta ) = 0.0031$ are used. The width of the microstrip is $w = 1.7$ mm, and the dimensions of the DGS etched in the ground plane are $a = 3$ mm, $b=3$ mm, $c=0.2$ mm, $g= 0.2$ mm, $e=2.6$ mm, and $d = 0.2$ mm. The crack to be sensed has a width $w_c=0.2$ mm, a length $l_c =4$ mm, and a depth $h_c=2$ mm. The metallic block and the ground plane are separated by an air gap $gap = 0.2$ mm. The EM simulated transmission and reflection coefficients of the sensor above an Aluminum block with and without a crack are plotted (using solid lines) in Fig. 12.

The results show a $15.5\%$ shift in the resonance frequency of the structure due to the presence of the crack in the metal surface, which shows $(15.5-14)/14$, i.e., just above $10\%$ improvement in sensitivity with respect to a single-ring CSRR with identical dimensions. Although small, the increase in the frequency shift confirms the method of sensitivity improvement, which is based on the theoretical analysis of the previous section. Also, note that the sensor benefits from 210% higher sensitivity with respect to the presented double-ring CSRR sensor.

It can be shown that the equivalent circuit model of the structure when the symmetry plane of the DGS is orthogonal to the loaded microstrip line is identical to the circuit model of a CSRR-loaded TL shown in Fig. 1d^18^. Therefore, it would be instructive to extract the parameters of the equivalent circuit model of the sensor based on the dumbbell-shape DGS and compare it with those of the sensor based on a single-ring CSRR. This is performed, and the extracted parameters are listed in Table 5. The table shows that when the DGS is over a faultless metal surface, all the extracted parameters are almost equal to those of the single-ring CSRR listed in Table 2. However, when the DGS is over a crack in the metal surface, the equivalent inductance *L* is increased by $55\%$. This is almost $20\%$ higher compared to the case of the single-ring CSRR. For comparison, the simulated scattering parameters of the equivalent circuit model with extracted parameters are plotted using dashed lines in Fig. 12.

**Table 5:** The extracted parameters for the sensor of Fig. 11 at two states; over the faultless and over the cracked metal surface..

**Parameter**	**Over faultless metal**	**Over cracked metal**	%
*L* (nH)	0.11	0.17	55.21
*C* (pF)	2.43	2.15	-11.61
$L_\ell$ (nH)	1.04	1.06	1.50
$C_c$ (pF)	0.30	0.32	3.71

**Figure 13:**
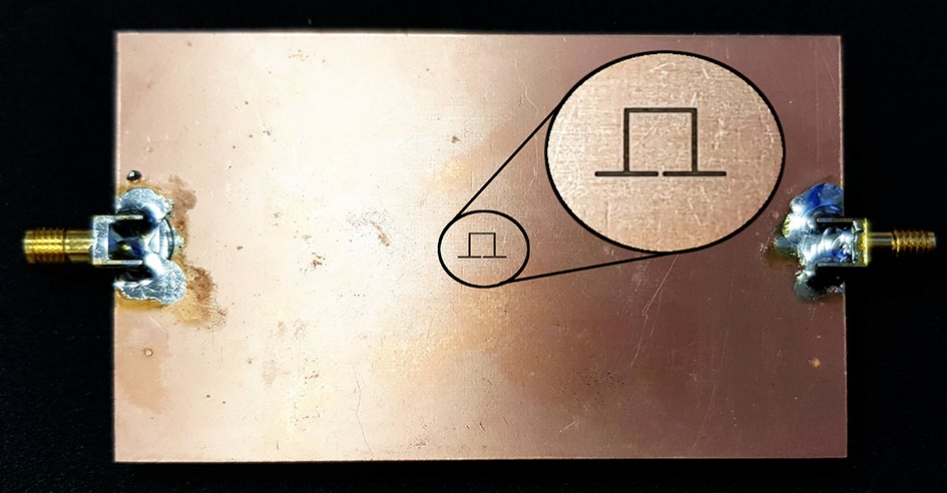
A photograph of the fabricated prototype of a crack sensor based on a folded dumbbell-shape DGS.

**Figure 14:**
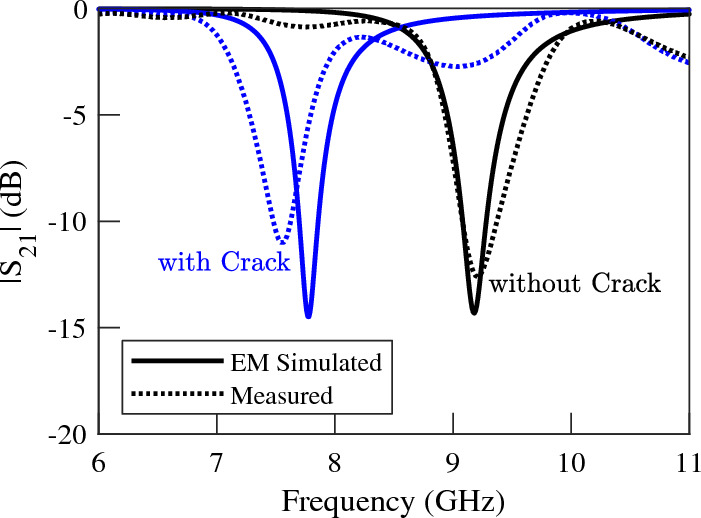
Measured (dotted lines) and simulated (solid lines) magnitude of the transmission coefficients of the sensor of Fig. 13 when the sensor is located above an Aluminum block with and without a crack.

Experimental validation of the concept and numerical results is conducted through the fabrication and measurement of a prototype of the proposed sensor based on dumbbell-shape DGS. A photograph of the fabricated prototype is presented in Fig. 13. All the dimensions and the utilized materials of the prototype correspond to the simulated structure. Measured and EM-simulated transmission coefficients of the structure over faultless and over cracked metal surfaces are compared in Fig. 14. Good agreement between the numerical and experimental results is observed.

### Detecting cracks with arbitrary orientation using DGS

It is important to note that the sensors presented in the previous subsections are only sensitive to cracks that are orthogonal to the inductive strip (bridge) of the resonator. This is because changes in the inductance $\Delta L$ of the resonators are mainly due to the penetration of the magnetic field and thus storage of magnetic energy in the interior volume of the crack. This conveniently happens when a narrow crack is aligned with the magnetic field lines of the CSRR (i.e. when the crack is normal to the CSRR magnetic wall). However, it is clear that the magnetic fields passing through a crack that is oriented along the symmetry plane of the CSRR (i.e. along the magnetic wall of the CSRR) are relatively small. Therefore, a CSRR is much less sensitive to cracks aligned to its symmetry plane. However, in this case, the stored magnetic energy in the volume of the crack can be increased by using a longer inductive strip. It will be shown in this section that this goal can be achieved by using a dumbbell-shaped DGS with larger defects.

**Figure 15:**
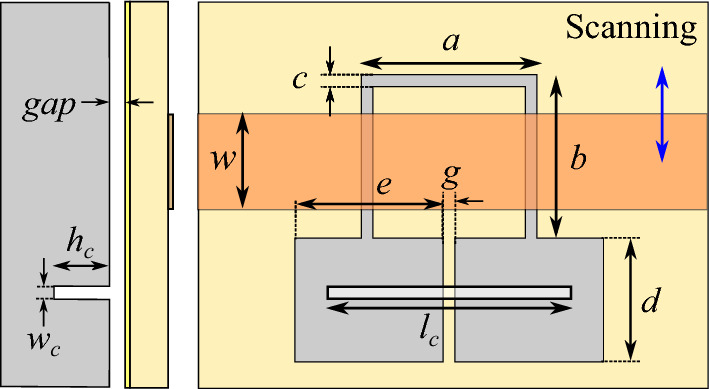
Illustrations of the top and side views of the proposed sensor based on a folded dumbbell-shape DGS for sensing cracks with arbitrary orientations.

An illustration of the proposed sensor is shown inComparison between the proposed Fig. 15. The sensor is identical to the sensor of the previous subsection, except that the dimensions of the rectangular defects, i.e. *e* and *d* are modified to increase the length of the inductive strip. In this structure, the dimensions of the defect can be optimized to achieve equal sensitivities to cracks in both parallel and orthogonal orientations. It is clear that such resonator can also detect cracks with an arbitrary orientation (i.e., with any angle between 0 and 90 degrees).

**Figure 16:**
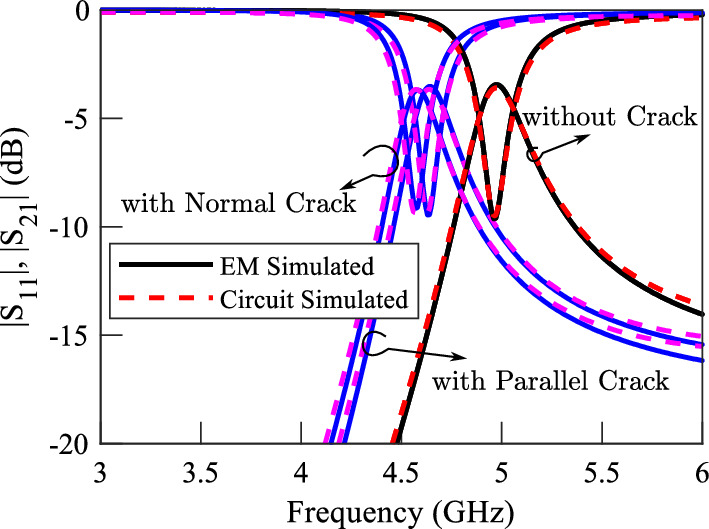
Simulated magnitude of the transmission and reflection coefficients of the sensor in Fig. 15 when the sensor is located above a faultless Aluminum block as well as above an Aluminum block with cracks in normal and parallel orientations.

For demonstration, the performance of a DGS with $e = d = 2.6$ mm, i.e., with square-shape defects is studied using full-wave EM simulations. For simulation purposes, the same substrate and microstrip line with identical dimensions to those in the previous sensors are used. The EM simulated scattering parameters of the sensor above a faultless metal surface as well as above a metal surface with a crack (1) normal, and (2) parallel to the symmetry plane of the DGS are plotted using solid lines in Fig. 16. The EM simulation results show a $5.4\%$ shift in the resonance frequency of the structure due to a crack normal to the symmetry plane of the DGS, and a $4.3\%$ shift in the resonance frequency due to a crack parallel to the symmetry plane of the DGS. The frequency shift due to cracks with arbitrary orientations is in the same range (not shown).

Again, the circuit presented in Fig. 1d is used to model the structure. The extracted parameters of the equivalent circuit model of the sensor when sensing a faultless metal and when sensing a crack orthogonal to the bridge are listed in Table 6. The parameters of the sensor when sensing a crack parallel to the bridge are listed in Table 7. The values show that the changes in the equivalent inductance *L* for cracks with both normal and parallel orientations are almost equal. For comparison, the simulated scattering parameters of the equivalent circuit model with extracted parameters are plotted using dashed lines in Fig. 16.

**Table 6:** The extracted parameters for the sensor of Fig. 15 for a crack normal to the symmetry plane of the DGS at two states; over the faultless and over the cracked metal surface..

Parameter	Over faultless metal	Over cracked metal	%
*L* (nH)	0.59	0.68	14.97
*C* (pF)	1.46	1.46	0.15
$L_\ell$ (nH)	0.93	0.93	0.27
$C_c$ (pF)	0.28	0.28	-1.12

**Table 7:** The extracted parameters for the sensor of Fig. 15 for a crack parallel to the symmetry plane of the DGS at two states; over the faultless and over the cracked metal surface..

Parameter	Over faultless metal	Over cracked metal	%
*L* (nH)	0.59	0.66	11.82
*C* (pF)	1.46	1.55	6.35
$L_\ell$ (nH)	0.93	0.92	-0.31
$C_c$ (pF)	0.28	0.29	1.79

**Figure 17:**
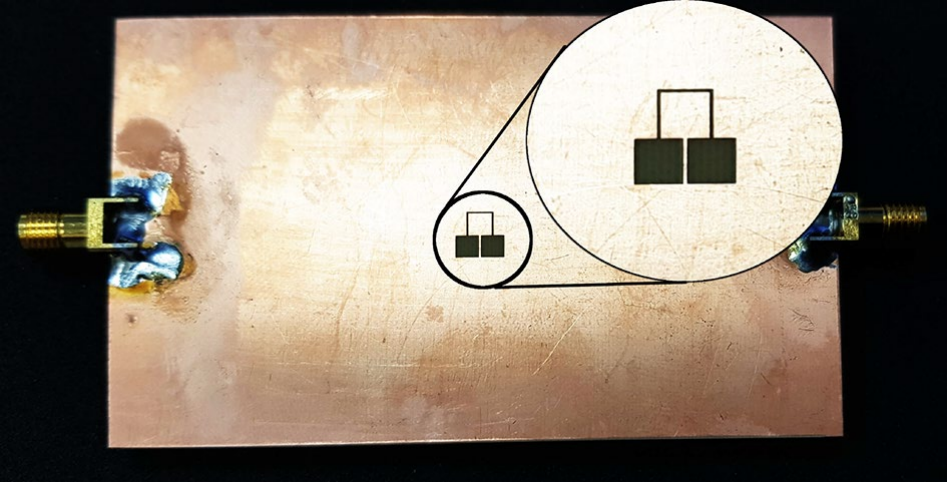
A photograph of the fabricated prototype of a crack sensor based on a folded dumbbell-shape DGS with square defected areas for sensing cracks with arbitrary orientations.

**Figure 18:**
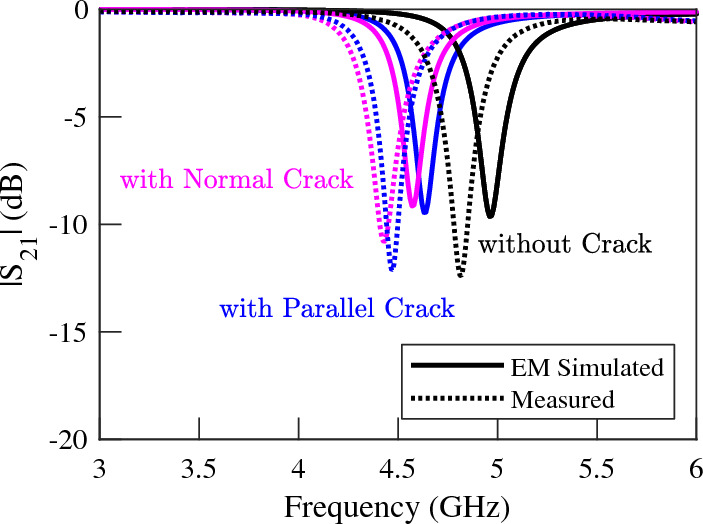
Measured (dotted lines) and simulated (solid lines) magnitude of the transmission coefficients of the sensor of Fig. 17 when the sensor is located above a faultless metal surface as well as a faulty metal surface with cracks in normal and parallel orientations.

For experimental validation, a prototype of the simulated sensor is fabricated and its performance is measured. A photograph of the fabricated prototypes is presented in Fig. 17. All the dimensions and the utilized materials of the prototype correspond to the simulated structure. Measured and EM-simulated transmission coefficients of the structure over faultless and over metal surfaces with normal and parallel cracks are shown in Fig. 18. The measurement results show a slight shift in the overall operating frequency of the sensor, which can be due to tolerances in the fabrication. However, the identical shift in the resonance frequency due to the crack in the measurements and simulations validates the proposed concept.

**Table 8:** Comparison between the proposed crack sensors and state-of-the-art sensors.

References	Technology	Operating frequency	Dimensions	Crack length *L*	Sensitivity $\Delta f_0/f_0$	Detecting cracks of
		(GHz)	($\lambda _g^2$)	(mm)	($\%$)	Arbitrary orientations
^11a^	Rectangular patch	2.485/2	0.66	Unlimited	2.1/3.42	Yes
^29^	CSRR-Loaded SIW	5.12	0.25	Unlimited	7.8	No
^27^	Dual-mode resonator	13.87	Not presented	10	0.72	No
^30b^	Double-ring CSRR	6.4	0.02	4	5	No
^32b^	Slotted double-ring CSRR	6.4	0.02	4	5	No
This work	Single-ring CSRR	9.57	0.044	4	14	No
This work	DGS (Fig. 11)	9.2	0.044	4	15.5	No
This work	DGS (Fig. 15)	5	0.012	4	5.4 /4.3	Yes

^a^Crack width $w_c=0.6$ mm.

^b^Re-simulated.

### Discussion

This subsection briefly compares the proposed metal crack sensors to the state-of-the-art crack sensors published in the literature. Detailed information of the comparison is listed in Table 8. The main figure of merits considered in this study are the sensor’s compactness, sensitivity, and capability of detecting cracks with arbitrary orientations. Since this study is focused on compact and low-profile sensors, bulky non-planar crack sensors such as those based on hollow waveguides are excluded from the comparison.

Regarding the compactness, note that the three sensors proposed in this work and those presented in^30,32^ have identical physical sizes. However, since they operate at different frequencies their electrical size is different. Nonetheless, the comparison shows that the sensors presented in this work are among the most compact crack sensors. The table also compares different sensors in terms of their sensitivity, i.e., their normalized frequency shift $\Delta f_0/f_0$ due to the presence of a crack with 0.2 mm width, 2 mm depth, and 4 mm length. As listed in the table, the cracks that are used to evaluate the sensors in^11,27,29^ are much longer. Therefore, the sensitivity of these sensors to a 4 mm long crack might be less than those reported in the table. Overall, the comparison shows that the sensors proposed in this research benefit from the highest sensitivities. Finally, as shown in the table, only two sensors are capable of detecting cracks with arbitrary orientations: The last sensor presented in this work and that of^11^. However, the sensor presented in^11^ is much larger than other sensors in the table. It also suffers from relatively low sensitivity. It should be noted that the sensitivity of this sensor has been evaluated based on a 0.6 mm wide crack with unlimited length.

**Figure 19:**
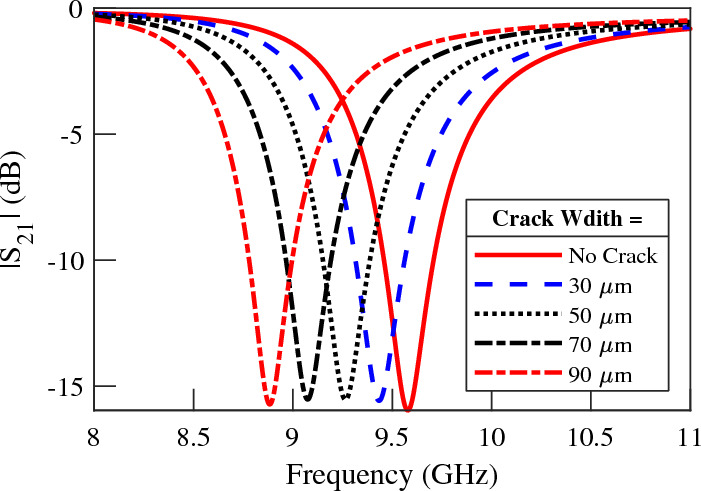
Simulated transmission coefficient of the single-ring CSRR sensor for different crack widths.

The resolution, i.e., the minimum crack size that can be reliably detected, is another important characteristic of the sensors. Note that the resolution of the proposed sensors is tied to the minimum detectable frequency shift. Therefore, to determine the resolution of the sensors, a minimum frequency variation of 100 MHz is assumed. Then, a parametric study on different crack widths and their influence on the frequency shift is conducted. The results of our investigation on the resolution of the single-ring CSRR sensor, depicted in Fig. 19, reveal that cracks as minute as $30$ µm induce a discernible frequency shift of 140 MHz. In short, under the premise of a minimum detectable frequency shift of 100 MHz, our proposed sensors achieve a resolution finer than $30$ µm. Importantly, it’s worth noting that the potential for achieving even higher crack-sensing resolutions exists if we can accurately determine smaller frequency shifts.

Overall, the results of the comparison suggest that the theoretical analysis and guidelines provided in this study were highly effective in designing microwave crack sensors with improved sensitivity or compact sensors with moderate sensitivity that are capable of detecting cracks with arbitrary orientations.

## Conclusion

The main objective of the current study was to provide a theoretical framework for the design of crack sensors with improved sensitivity. With this aim, sensitivity analysis of various types of microwave resonators from the equivalent circuit perspective has been presented. On that basis, several essential questions regarding the type and the parameters of a microwave resonator that leads to sensors with improved sensitivity have been addressed. To gain a deeper insight, the questions have been also treated from the EM field perspective, and guidelines for the design of sensors with high sensitivity to cracks with sub-millimeter dimensions have been drawn up. For demonstration, the gained insight has been used to design two extremely simple but at the same time, highly sensitive crack sensors based on single-ring CSRR and folded dumbbell-shaped DGS resonators. Furthermore, it has been shown that the shape of the folded dumbbell-shaped DGS resonator can be modified to create a new sensor that can sense cracks of arbitrary orientations. The behavior of the three proposed sensors while sensing faultless and faulty metal surfaces has been thoroughly studied through the EM and circuit simulations of the structures. Moreover, the designed sensors have been fabricated and the numerical results have been validated through the measurement of the fabricated prototypes.

## Data Availability

The data produced and analyzed during the current study are available from the corresponding author on reasonable request.
